# Effect of Human Flavin-Containing Monooxygenase 3 Polymorphism on the Metabolism of Aurora Kinase Inhibitors

**DOI:** 10.3390/ijms14022707

**Published:** 2013-01-28

**Authors:** Gianluca Catucci, Andrea Occhipinti, Massimo Maffei, Gianfranco Gilardi, Sheila J. Sadeghi

**Affiliations:** Department of Life Sciences and Systems Biology, University of Torino, Via Accademia Albertina 13, Torino 10123, Italy; E-Mails: gianluca.catucci@unito.it (G.C.); andrea.occhipinti@unito.it (A.O.); massimo.maffei@unito.it (M.M.); gianfranco.gilardi@unito.it (G.G.)

**Keywords:** phase I, drug metabolism, FMO3, polymorphism, enzyme kinetics, VX-680, PHA-739358, LC-MS

## Abstract

Aurora kinases were recently identified as a potential target in anticancer therapy and, amongst their available inhibitors, Tozasertib (VX-680) and Danusertib (PHA-739358) have been indicated as possible substrates of human flavin-containing monooxygenase 3 (hFMO3). Here we report the *in vitro* rate of oxidation of these drugs by wild-type hFMO3 and its polymorphic variant V257M. The conversion of Tozasertib and Danusertib to their corresponding metabolites, identified by LC-MS, by the purified wild-type and V257M hFMO3 show significant differences. In the case of Tozasertib, the V257M variant shows a catalytic efficiency, expressed as *k*_cat_/*K*_m_, similar to the wild-type: 0.39 ± 0.06 min^−1^μM^−1^ for V257M compared to 0.33 ± 0.04 min^−1^μM^−1^ for the wild type. On the other hand, in the case of Danusertib, V257M shows a 3.4× decrease in catalytic efficiency with *k*_cat_/*K*_m_ values of 0.05 ± 0.01 min^−1^μM^−1^ for V257M and 0.17 ± 0.03 min^−1^μM^−1^ for the wild type. These data reveal how a simple V257M substitution ascribed to a single nucleotide polymorphism affects the *N*-oxidation of relevant anticancer drugs, with important outcome in their therapeutic effects. These findings demonstrate that codon 257 is important for activity of the hFMO3 gene and the codon change V to M has an effect on the catalytic efficiency of this enzyme.

## 1. Introduction

Human flavin-containing monooxygenase 3 (hFMO3) is the second most important Phase I drug metabolising enzyme after cytochromes P450. It is expressed in the adult human liver where it catalyses the NADPH-dependent *N*- and *S*-oxidation of drugs and xenobiotics [1]. Previously it has been reported that there is a 10- to 20-fold inter-individual difference in *FMO* expression that may contribute to drug response [2–4]. Furthermore, the existence of polymorphisms affecting hFMO3-dependent metabolism has also been documented [5], with several studies showing reduced activity with some common variants.

Several polymorphic variants of hFMO3 have been identified among which V257M (rs2266782), E158K (rs2266780), and E308G (rs1736557) are the most commonly distributed. Inter-individual and inter-ethnic variability in hFMO3 expression and enzyme activity is primarily due to genetics, therefore, making it possible to correlate hFMO3 allelic variants to drug-induced side effects or drug response. It has been previously shown that minor allele frequencies of E158K, V257M, and E308G vary greatly among different ethnic populations with distinct genetic backgrounds [6]. Moreover, allelic variation within the hFMO3 gene can also influence a person’s drug responsiveness [7] with important differences in the therapeutic response of life-saving drugs such as anticancer drugs.

Aurora A, Aurora B, and Aurora C are serine-threonine kinases that regulate mitotic events. The first two are the best studied and were assigned different roles in mitosis [8]: Aurora A was found to be over-expressed in many human tumors like breast, colorectal, and ovarian cancer [8], and Aurora B is also over-expressed in various types of cancer [9,10]. Specific inhibitors of Aurora kinases, such as VX-680, MLN8054, AZD1152, R766, R763, and PHA-739358, have been designed and screened for their inhibitory activity in cancer events [11].

The VX-680 inhibitor, also known as Tozasertib, was originally discovered by Harrington and co-workers [12], and it is an inhibitor of Aurora-A, Aurora-B, and Aurora-C kinases with inhibition constants (Ki_app_) of 0.6, 18 and 4.6 nM, respectively [12,13].

Rational design of the pyrrolopyrazole identified from the combinatorial expansion of the 1,4,5,6-tetrahydropyrrolo[3,4-*c*]pyrazoles bound to the pocket of the target led to the discovery of the PHA-739358 inhibitor, also called Danusertib [14,15]. This compound was also found to inhibit several tyrosine kinases such as Abl, Ret, Trk-A, and FGFR-1 that are involved in many types of cancer such as leukemia, thyroid, prostate, and breast carcinoma [14,15].

Tozasertib is metabolised into the *N*-desmethyl product by CYP3A4 and CYP2C8, and into the *N*-oxide product by CYP3A4 and hFMO3, as previously demonstrated by Ballard and co-workers [16]. Their study highlighted the combined contribution of hFMO3 and P450 3A4 to the formation of the *N*-oxide metabolite in human liver microsomes.

Knowledge of genetic polymorphism and its possible related functional changes is increasingly considered critical for both drug discovery and drug development [17,18]. Since Tozasertib and Danusertib have been indicated as substrates of the drug metabolizing flavin-containing monooxygenases, in this work we measure the kinetic parameters of the wild type hFMO3 (WT) and V257M polymorphic variant with these Aurora kinase inhibitors.

## 2. Results and Discussion

In order to determine the kinetic parameters related to the metabolism of Tozasertib and Danusertib by hFMO3, both WT and the V257M polymorphic variant were heterologously expressed in *Echerichia coli* and purified as previously described [19–22]. Both proteins showed a single band on SDS-PAGE gel with a yield of around 12 mg of protein per litre of culture. A molar ratio of more than 91% holo protein was determined spectrophotometrically [21] for both proteins. Positive control reactions, with benzydamine and methimazole as substrates, were performed to confirm the catalytic activities of the enzyme preparations.

We have chosen the V257M and not the other two common SNPs (single nucleotide polymorphism) due to the fact that residue 257 is located in the “insert” region of the *hFMO3* gene which is absent in the bacteria and yeast FMO amino acid sequences [23]. As a consequence, this “insert” region is not present in the two published crystal structures of FMO and, therefore, the exact location of this amino acid in relation to the active site of this enzyme can only be hinted at by *ab initio* modeling. We have previously generated a model of hFMO3 [19], however in our model the location of residue 257 is not very close to the FAD catalytic center, similar to the modeling results of Rettie and colleagues [23].

In a previous study we had shown the turnover of the WT hFOM3 in the presence of both Danusertib and Tozasertib using HPLC analysis [21]. In the current report, the metabolites of the enzymatic reaction of both WT and V257M polymorphic variant with Danusertib and Tozasertib were identified by LC-MS. In each case, the occurrence of the enzymatic oxidative reaction was confirmed by the molecular shift of 16 atomic mass units (amu) of both substrates. The oxidation sites were confirmed through comparison with LC-MS fragmentation profiles of substrates and observed metabolites.

### 2.1. Tozasertib Metabolism by hFMO3

**Figure 1:**
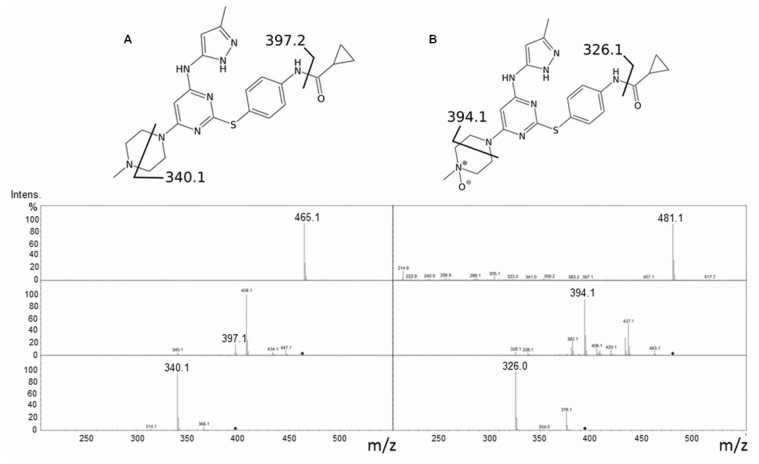
(**a**) Fragmentation profile of Tozasertib showing the molecular ion [M + H]^+^ 465.2 and subsequent 397.2 and 340.0 in MS_(3)_ as cleavage of C_4_O group and pyridine ring, respectively; (**b**) Tozasertib-*N*-oxide fragmentation showing the molecular ion [M + H]^+^ 481.2, the cleavage of *N*-oxide pyridine ring (394.1) first and the cleavage of C_4_O in the last fragmentation step.

In 2007, Ballard and co-workers [16] identified the metabolites of Tozasertib generated in human hepatocytes that were mainly the *N*-oxide and the desmethyl products. They went on to characterize the hepatic enzymes responsible and found that hFMO3 appreciably (50%–60%) contributed to the *N*-oxidation of Tozasertib in human liver microsomes together with cytochrome P450 3A4. Following in their footsteps, we investigated the metabolism of Tozasertib using the purified WT hFMO3, and identified the metabolite by LC-MS. The suggested fragmentation profile of Tozasertib (Figure 1A) includes the molecular ion [M + H]^+^ 465.2 and subsequent 397.2 and 340.0 in MS_(3)_ as cleavage of C_4_O group and pyridine ring, respectively. On the other hand, Tozasertib-*N*-oxide fragmentation (Figure 1B) shows the molecular ion [M + H]^+^ 481.2, the cleavage of *N*-oxide pyridine ring (394.1) first and the cleavage of C_4_O in the last fragmentation step.

The same enzymatic reactions followed by LC-MS analyses were carried out for the V257M polymorphic variant in order to test whether the variant was not only capable of metabolising Tozasertib, but also resulted in the same *N*-oxide metabolite.

### 2.2. Danusertib Metabolism by hFMO3

The major route of Danusertib metabolism involves the formation of *N*-oxide product by the action of hFMO3 forming an inactive metabolite. A possible influence of a SNP in the hFMO3 gene, on the metabolism of Danusertib, was proposed by Steeghs and co-workers in 2010 [24] when they showed a different clearance of the drug from the body in a patient heterozygous for V257M. Since the latter correlation between Danusertib pharmacokinetics and genetic variation was based on the data from a single patient, we thought the role of V257M in the metabolism of Danusertib warranted further investigation and decided to check the kinetics of Danusertib metabolism with the purified hFMO3 V257M polymorphic variant. To this end, initially *in vitro* experiments were set up for the identification of the *N*-oxide product by LC-MS and subsequently followed by the determination of the kinetic parameters of the enzymatic reactions.

**Figure 2:**
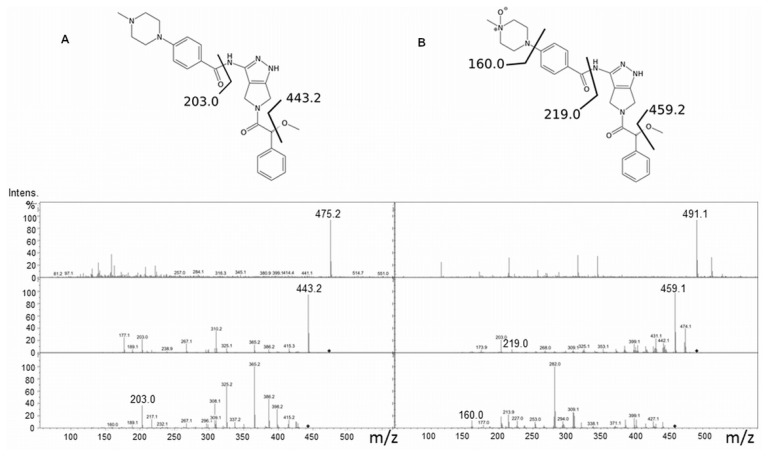
(**a**) Fragmentation profile of Danusertib showing the molecular ion [M + H]^+^ 475.2 and subsequent 443.2 as loss of the common methoxy group [M-CH_3_OH]^+^ at the first fragmentation step; (**b**) Fragmentation profile of Danusertib *N*-oxide showing the molecular ion [M + H]^+^ 459.2 as loss of the common methoxy group [M-CH_3_OH]^+^ at the first fragmentation step.

For the LC-MS experiments, purified WT and V257M hFMO3 were incubated with NADPH and Danusertib and the enzymatic reaction terminated after 10 min, and the resulting metabolites identified as described in the experimental section. Mass spectrometry analysis for Danusertib and its *N*-oxide showed, at the first fragmentation step, the loss of the common methoxy group [M-CH_3_OH]^+^ (443.2 and 459.2 for Danusertib and its *N*-oxide). Their MS_(2)_ fragmentation generates ion 203.0 for Danusertib and the 16 amu-shifted fragment at 219.0 for the *N*-oxide product (Figure 2A,B). Moreover, the latter showed a fragmentation ion from *N*-oxide pyridine ring at 160.0 that limits the *N*-oxidation site to the quaternary amine on the pyridine ring.

### 2.3. Kinetic Measurements of hFMO3 with Aurora Kinase Inhibitors

**Figure 3:**
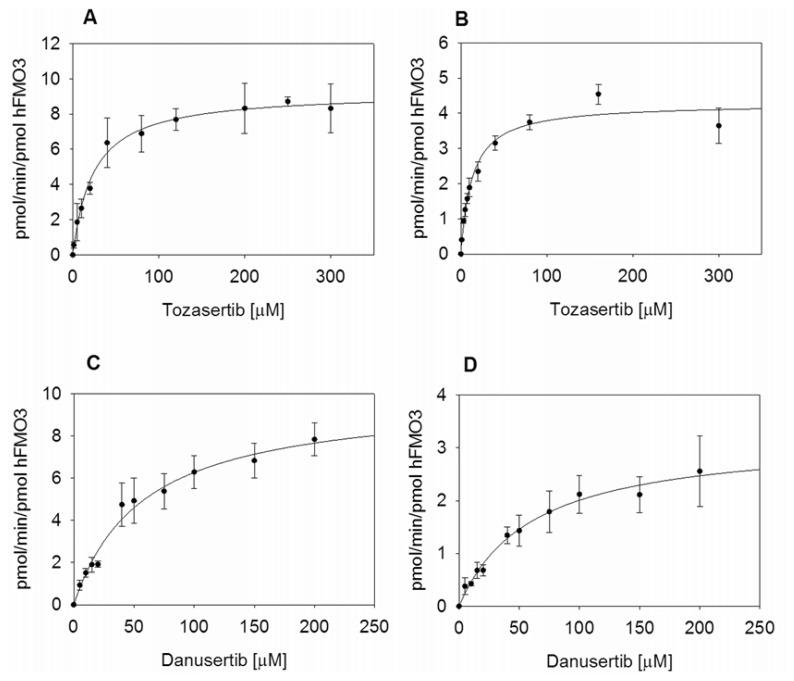
(**a**) Michaelis-Menten kinetics for the formation of the *N*-oxide metabolite of Tozasertib and Danusertib with wild type (**A** and **C**, respectively), and V257M (**B** and **D**, respectively) hFMO3. Reactions were carried out with 0.17 μM of each purified enzyme in 50 mM potassium phosphate buffer pH 7.4 and 0.5 mM NADPH in the presence of increasing amounts of Tozasertib (0–300 μM) and Danusertib (0–200 μM), at 37 °C for 10–15 min.

Enzymatic reactions were carried out with the purified WT and V257M polymorphic variant in the presence of NADPH and increasing amounts of each substrate as reported in the experimental section. Kinetic parameters for *N*-oxygenation of Tozasertib were determined by nonlinear regression analysis and showed a *K*_m_ of 23.8 μM, a *k*_cat_ of 9.3 min^−1^ in the case of the wild-type enzyme and a *K*_m_ value of 12.9 μM, a *k*_cat_ of 4.3 min^−1^ for the polymorphic variant V257M. The *k*_cat_ and *K*_m_ values for V257M were approximately half of that of the wild-type (Figure 3A,B) and the *k*_cat_ for the wild-type enzyme was at least twice as much of the *k*_cat_ for V257M. When the ratios of *k*_cat_/*K*_m_ are compared, both enzymes seem to be equally efficient in transforming Tozasertib into the *N*-oxide product. The *K*_m_ values measured are slightly higher when compared to the data published by Ballard and co-workers [16], probably because they specifically address the substrate binding to hFMO3 and do not consider the role of cytochrome P450 3A4 that also contributes to the overall *N*-oxide metabolite of Tozasertib. Moreover, for Tozasertib the V_max_ of wild type hFMO3 was 536 pmol/min/mg FMO3 while V257M hFMO3 V_max_ resulted in 248 pmol/min/mg FMO3 (Table 1) in good agreement with the data published by Ballard and co-workers [16].

Kinetic parameters for *N*-oxygenation of Danusertib were also determined by nonlinear regression analysis, and showed a *K*_m_ of 57.3 μM, a V_max_ of 0.57 nmol min^−1^ mg FMO3, and *k*_cat_ of 9.9 min^−1^ for the wild-type enzyme and a *K*_m_ of 60.1 μM, a V_max_ of 0.18 nmol min^−1^ mg FMO3 and *k*_cat_ of 3.2 min^−1^ for V257M (Figure 3C,D). The *K*_m_ value for the polymorphic variant V257M was very similar to that of the wild-type indicating that Danusertib binding is similar in the two proteins. More interestingly the V_max_ in the case of V257M was approximately three times less than the wild-type (Table 1), showing a decreased turnover rate that yields a significantly lower turnover number. The *k*_cat_/*K*_m_ ratio is three times higher for the wild type hFMO3, which means that the variant V257M is less efficient in metabolizing Danusertib.

## 3. Experimental Section

### 3.1. Reagents

FAD, acetonitrile, methanol, NADPH, Verapamil and salts were purchased from Sigma-Aldrich (Milan, Italy). VX-680 and PHA-739358 were purchased from Aurogene (Rome, Italy).

### 3.2. Recombinant FMO3 Protein Preparation

WT hFMO3 was cloned in the expression vector pJL2 using the two restriction enzymes, XbaI and HindIII, as previously reported [19,21]. QuikChange Site-Directed Mutagenesis Kit (Stratagene, Torino, Italy) was used to obtain the V257M polymorphic variant using the following two primers (mutation site in bold):

5′ CT GAC TGG TTG TAC **ATG** AAG CAG ATG AAT GC 3′

5′ GC ATT CAT CTG CTT **CAT** GTA CAA CCA GTC AG 3′

Subsequently the presence of the correct mutation was confirmed by sequencing the entire clone.

Wild type and V257M hFMO3 were heterologously expressed in *E. coli* JM109 cells and grown 24 h post-induction. Both proteins were purified from the membrane fractions using Ni affinity chromatography. Spectra of the fractions eluted with 40 mM histidine were recorded using a diode array HP-8453E spectrophotometer. FAD-containing fractions with the characteristic absorption peaks at 375 and 442 nm were pooled and exchanged with storage buffer (100 mM potassium phosphate buffer pH 7.4, 20% glycerol and 1 mM EDTA) by 30 kDa cutoff Amicon membranes (Millipore, Billercia, MA, USA) and stored at −20 °C.

The concentration of hFMO3 was determined by spectroscopy with the peak absorbance at 450 nm and an extinction coefficient of 11900 M^−1^ cm^−1^. This value was also used for determination of the enzyme concentration under non-denaturing conditions. The yield of the purified hFMO3 protein was determined using both absorbance at 280 nm (calculated extinction coefficient of 87520 M^−1^ cm^−1^) and Bradford assay.

### 3.3. Enzyme Assays

*N*-oxygenation of Tozasertib and Danusertib was evaluated by incubating a mixture of 0.17 μM of purified enzyme in 50 mM potassium phosphate buffer (pH 7.4), 0.5 mM NADPH, and increasing amounts of substrate in a final volume of 0.20 mL. The enzyme and NADPH were mixed first and the reaction was initiated by the addition of substrate, preventing thermal degradation of the enzyme. Incubations were carried out at 37 °C for 10 min in the case of Danusertib, and 15 min in the case of Tozasertib: the linearity of product formation was confirmed with purified hFMO3 preparations for 20 min. The incubations were terminated by the addition of 0.10 mL of ice-cold methanol and 150 μL verapamil as internal standard (IS).

### 3.4. LC-MS Analyses of Danusertib, Tozasertib and Corresponding N-oxides

Prior to LC-MS analysis the reaction mixtures were filtered through a Microcon centrifugal membrane with 3 kDa cutoff (Millipore, Billercia, MA, USA). Substrates and their metabolites were separated using Agilent 1200 HPLC (Agilent Technologies Inc., Santa Clara, CA, USA) equipped with a narrow bore reversed-phase column Zorbax Eclipse XDB-C18, 2.1 × 150 mm, 3.5 μm (Agilent Technologies, Santa Clara, CA, USA) and using a binary solvent system composed of double distilled water with 0.01% formic acid (A), and acetonitrile (ACN) with 0.1% formic acid (B) at flow rate of 0.2 mL min^−1^. The initial mobile phase was composed of 20% of solvent B and it was increased to 70% over a period of 3 min and kept 4 min for isocratic separations of substrate, metabolite and internal standard (IS). Finally, the organic solvent was increased to 100% in 30 s for washing the column. Before follower injection, initial mobile phase was re-established for 10 min.

Prior to MS detection, the column eluate was monitored on-line with UV detection at 250 nm wavelength for substrates and their metabolites, and 280 nm for IS. The MS analyses were made by a 6330 Series Ion Trap Mass Spectrometer (Bruker Daltonik GmbH, Bremen, Germany) equipped with an electrospray ionization source.

Analyses for fragmentation spectra were conducted by ESI-MS/MS operating in full scan from 50 to 900 *m*/*z*. Spectra were acquired in positive mode with ion spray voltage at 1.2 kV, nebulizer gas (N^2^) at 15 psi and 5 L min^−1^, dry temperature at 325 °C, and 1.00 V fragmentation amplitude.

For quantitative purpose, standards and samples were analysed by LC-ESI-MS/MS in MRM mode using the above-indicated parameters. The monitored mass transitions were m/s 465.2→408.1, 481.2→437.1, 475.2→443.2, 491.2→459.2 and 455.3→303.1 for Tozasertib, Tozasertib-*N*-oxide, Danusertib, Danusertib-*N*-oxide and Verapamil, respectively. Spectral data were processed using data analysis software of 6300 Series Ion Trap LC/MS 4.0 (Bruker Daltonik GmbH, Bremen, Germany). Metabolite formation was quantified by comparing peak area ratios (metabolite/internal standard) of four data sets of incubations for each Michaelis-Menten curve to ratios obtained from a standard curve containing known amounts of the internal standard verapamil.

## 4. Conclusions

In conclusion, Tozasertib is metabolised by hFMO3 to the expected *N*-oxide. The V257M polymorphic variant displays little difference in the *N*-oxidation of this drug, so since cytochrome P450 3A4 and hFMO3 appear to be important in the latter reaction [16] the inter-individual variations related to the metabolism of this drug can only be due to genetic or environmental factors influencing the activity of cytochrome P450 3A4. On the other hand, Danusertib is also metabolised by hFMO3 to the corresponding *N*-oxide, but its metabolism is markedly influenced by the V257M polymorphism showing a 3.4 times lower catalytic efficiency compared to the wild type enzyme implying that inter-individual variations of the hFMO3 gene can result in decreased activity towards this drug.

Finally, the V257M substitution of hFMO3 represents a good example of how this SNP can influence the *N*-oxidation of relevant anticancer drugs. These findings demonstrate the need for further research into the consequences of flavin-containing monooxygenase genetic polymorphism in drug metabolism.

## Figures and Tables

**Table 1:** Kinetic analysis of Tozasertib and Danusertib metabolism by WT and V257M polymorphic variant of hFMO3.

	WT hFMO3				V257M hFMO3			

	*K*_m_	V_max_	*k*_cat_	*k*_cat_/*K*_m_	*K*_m_	V_max_	*k*_cat_	*k*_cat_/*K*_m_
	μM	nmol/min/mg FMO3	min^−1^	min^−1^ μM^−1^	μM	nmol/min/mg FMO3	nmol/min/mg FMO3	min^−1^ μM^−1^
Tozasertib	23.8 ± 3.4	0.54 ± 0.02	9.3 ± 0.3	0.39 ± 0.06	12.9 ± 1.3	0.25 ± 0.01	4.3 ± 0.1	0.33 ± 0.04
Danusertib	57.3 ± 8.3	0.57 ± 0.03	9.9 ± 0.6	0.17 ± 0.03	60.1 ± 11.4	0.18 ± 0.01	3.2 ± 0.3	0.05 ± 0.01
